# Intensive care photoplethysmogram datasets and machine-learning for blood pressure estimation: Generalization not guarantied

**DOI:** 10.3389/fphys.2023.1126957

**Published:** 2023-03-02

**Authors:** Guillaume Weber-Boisvert, Benoit Gosselin, Frida Sandberg

**Affiliations:** ^1^ Department of Electrical and Computer Engineering, Université Laval, Quebec, QC, Canada; ^2^ Department of Biomedical Engineering, Lund University, Lund, Sweden

**Keywords:** blood pressure estimation, BP estimation, photoplethysmography, mimic, UCI, PPG-BP, PPG datasets, intensive care datasets

## Abstract

The large MIMIC waveform dataset, sourced from intensive care units, has been used extensively for the development of Photoplethysmography (PPG) based blood pressure (BP) estimation algorithms. Yet, because the data comes from patients in severe conditions—often under the effect of drugs—it is regularly noted that the relationship between BP and PPG signal characteristics may be anomalous, a claim that we investigate here. A sample of 12,000 records from the MIMIC waveform dataset was stacked up against the 219 records of the PPG-BP dataset, an alternative public dataset obtained under controlled experimental conditions. The distribution of systolic and diastolic BP data and 31 PPG pulse morphological features was first compared between datasets. Then, the correlation between features and BP, as well as between the features themselves, was analysed. Finally, regression models were trained for each dataset and validated against the other. Statistical analysis showed significant $\left(p<0.001\right)$ differences between the datasets in diastolic BP and in 20 out of 31 features when adjusting for heart rate differences. The eight features showing the highest rank correlation $\left(\left|\rho \right|\;>\;0.40\right)$ to systolic BP in PPG-BP all displayed muted correlation levels $\left(\left|\rho \right|\;<\;0.10\right)$ in MIMIC. Regression tests showed twice higher baseline predictive power with PPG-BP than with MIMIC. Cross-dataset regression displayed a practically complete loss of predictive power for all models. The differences between the MIMIC and PPG-BP dataset exposed in this study suggest that BP estimation models based on the MIMIC dataset have reduced predictive power on the general population.

## 1 Introduction

Hypertension is one of the greatest challenges to public health of our time. According to the Centre for Disease Control, 47% of the adult population in the United States suffer from hypertension, and only 24% of those with hypertension have their condition under control (Centers for Disease Control and Prevention, 2022). Hypertension is an independent risk factor for cardiovascular diseases such as heart attack, stroke, and kidney disease, and ranks second amongst the preventable causes of death in the U.S., trailing cigarette smoking only (US Department of Health and Human Services, 2003; Kochanek et al., 2019; Danaei et al., 2009). It is now widely accepted that home blood pressure (BP) monitoring and ambulatory BP monitoring are much better at predicting risks associated with hypertension than in-clinic BP measurements (Ogedegbe and Pickering, 2010), with night time BP increasingly seen as an important risk determinant (Hansen et al., 2011; Gehring et al., 2018). Devices presently used for home BP monitoring utilize an inflatable cuff, which only provides intermittent readings instead of presenting the entire dynamic range and patterns of BP fluctuations. Moreover, the discomfort caused by cuff inflation is particularly problematic for nocturnal BP measurement, as it can disturb sleep and thereby interfere with measurements (Solà and Delgado-Gonzalo, 2019).

Photoplethysmography (PPG) based BP estimation shows promises to be a low-cost and convenient technique that enables wearable designs and has the potential to replace cuff-based devices (Elgendi et al., 2019). However, the lack of open access, standardized PPG datasets for training and testing BP estimation algorithms is an obstacle to researchers in the field. Most studies are based on private databases where composition of the data and methods of acquisition vary considerably, making a direct comparison between the published BP estimation algorithms impossible (Solà and Delgado-Gonzalo, 2019).

At the time of writing, several public datasets that include BP and PPG signal are available. There are two large datasets sourced from intensive care and surgical units: the Multiparameter Intelligent Monitoring in Intensive Care II (MIMIC) Waveform Dataset (Saeed et al., 2011) from the Massachusetts Institute of Technology, released on PhysioNet (Goldberger et al., 2000) in 2011, and the VitalDB from the Seoul National University Hospital (Lee et al., 2022) released in 2017. Several smaller datasets also exist, often with a focus on a specific condition. A few examples are: The University of Queensland Vital Sign Dataset (Liu et al., 2012), a 32 patient dataset focusing on anaesthesia acquired at the Royal Adelaide Hospital in Adelaide, Australia, released in 2012; the Bed-Based Ballistocardiography Dataset (Carlson et al., 2020), a 40 patient dataset from the Kansas State University, released at the end of 2020; and the PPG-BP dataset (Liang et al., 2018), a 219 patients dataset from the Guilin People’s Hospital, released in 2018, with a focus on the screening of cardiovascular diseases (CVD) from PPG.

The PPG-BP dataset can be considered a middle ground among the available datasets. It contains 657 short PPG segments three for each of the 219 patients and recorded at rest under controlled experimental conditions. Each patient is associated with a single BP measurement, as well as patient biometric data and diagnosed CVD, if any. In contrast, MIMIC contains more than 25,000 records of variable length and varying measurement types, at times including PPG and arterial blood pressure (ABP). The data was acquired from bedside monitoring devices at intensive care units (ICU), including surgery and cardiac care units, at the Beth Israel Deaconess Medical Center in Boston, United States. Among all the public datasets, MIMIC has been available the longest and has been used the most extensively in the field of BP estimation. The other datasets have seen little use in comparison, and some are not well suited for developing and validating BP estimation algorithms due to the limited number of subjects, the special conditions of data collection and the sporadicity of BP measurements.

MIMIC has been used in many BP estimation studies. Kachuee et al. used a sample of 3,663 records from 942 subjects to estimate systolic blood pressure (SBP) using 10 PPG and ECG morphological features. Their best results were a mean absolute error (MAE) of 11.17 mmHG without calibration and 8.21 mmHG with calibration, using AdaBoost for regression (Kachuee et al., 2017). In 2020 Hasanzadeh et al. used a sample of about 1,000 subjects to estimate SBP from one spectral and 18 morphological features using PPG only. Their best results were obtained with AdaBoost regression, giving a MAE of 8.22 mmHg (Hasanzadeh et al., 2020). In 2021, a subset of 200 subjects has been used by Esmaelpoor et al. to compare of 56 machine-learned features generated by convolutional neural network (CNN) against a set of 27 frequently used morphological features from PPG and ECG. Eight regression methods were tested and the best results were obtained with squared exponential Gaussian regression or Gaussian process regression depending on the test parameters, providing SBP with a MAE under 6 mmHg using morphological features, and under 3.5 mmHg using machine-learned features (Esmaelpoor et al., 2021). As in this last example, the dataset has been used many times with pulse transit time and pulse arrival time algorithms, despite that variability in the ECG sampling time makes it unsuitable for transit and arrival time calculation (Elgendi et al., 2019). The breadth and variable quality of the dataset also resulted in uneven sampling by researchers, and as such hardly makes performance comparison easier, even between two studies using it. A more serious concern is the frequently mentioned hypothesis that because the data is sourced from ICU, with patients having received medication and being in varied critical conditions, the MIMIC population may exhibit abnormalities or a different relation between PPG and BP than would be seen in a more controlled setting (Kachuee et al., 2017; Hasanzadeh et al., 2020; Chao et al., 2021), casting doubt on the validity of results beyond the dataset itself.

The aim of this study is to evaluate if the relationship between PPG pulse characteristics and BP in MIMIC is truly different from that in data acquired under controlled conditions. To achieve this goal a subset of MIMIC was compared to PPG-BP in a two-step approach. First, a statistical comparison of the datasets was performed. It comprised comparing the distribution of features characterizing PPG pulse morphology, as well as comparing the correlation between features on each dataset. Second, the correlation between BP and features was compared between datasets to see if similar morphological variations could be observed on both datasets in relation to BP changes. To illustrate the implication of the differences between the datasets, Support Vector Regression models were trained on each dataset and their cross-validated performance on the training set were compared to their performance on the other dataset, in order to assert whether predictive powers were retained.

## 2 Materials and methods

### 2.1 Datasets

A subset of the MIMIC database, prepared especially for BP estimation by Kachuee. (2015), is used in this study. Because it is hosted by the University of California, Irvine, the subset is sometimes called the “UCI” dataset, which will be used hereafter. This subset, which excludes segments with missing signals and abnormal values from MIMIC, contains 12,000 records of lengths varying between 8 s and 10 min. Each record is sampled at 125 Hz and contain fingertip PPG, electrocardiogram (ECG), and instantaneous ABP. No additional information about the subjects is provided, and the devices used for data acquisition are not specified.

The PPG-BP dataset contains 657 fingertip PPG segments from 219 subjects of 21–86 years of age with an average of 57 ± 16 years. Each segment has a duration of 2.1 s and a sampling rate of 1 kHz. A single SBP and diastolic blood pressure (DBP) measurement is provided for each subject, as well as the sex, age, height, weight, heart rate, and disease records. The PPG signal was recorded through an SMPLUS SEP9AF-2 sensor connected to a Texas Instrument MSP430FG4618 microcontroller, with a hardware filter bandpass of 0.5–12 Hz. The BP measurements were taken with an Omron HEM-7201 upper arm BP monitor. While also sourced from hospital patients, the PPG-BP data does not come from ICU units and was acquired under controlled conditions following an experimental protocol. Data acquisition was conducted in private, following a relaxation and adaptation period of 10 min, with the patients sitting in an office chair and their arms resting on a desk. The same acquisition devices were used for all subjects. Furthermore, a screening process excluded patients diagnosed with diseases other than cardiovascular diseases and diabetes. The data was also screened for abnormal and missing values, while a consistent signal quality was ensured by computing a signal quality index and excluding subjects with low values (Liang et al., 2018).

### 2.2 Pre-processing

All signal processing was done in Python and references to functions are, otherwise noted, part of the standard library or of the *SciPy* scientific library (Virtanen et al., 2020).

For UCI, five evenly spaced segments of a duration of 5 seconds were first extracted from each of the records in the dataset. Records shorter than 25 s were rejected. SBP and DBP were extracted from the continuous ABP signal by averaging all the peak values in the sequence, using function *find_peaks*. Records with less than three ABP peaks, due to non-pulsatile ABP segments, were rejected. Even though the UCI dataset had already been pre-processed to eliminate invalid or excessively noisy signals found in MIMIC, signal segments with movement artefacts, as well as sequences with large variations in pulsatile amplitude remained. To eliminate those issues and ensure coherence between the datasets, the following pre-processing steps were applied to both UCI and PPG-BP. First, all segments had their mean removed and were then filtered using a 0.7–12 Hz zero-phase fourth order Butterworth bandpass filter.

Three screening criteria were created to identify the remaining problematic segments. Any segment satisfying one of the conditions was rejected. The first criterion excluded signal segments with very rapid changes associated with signal artefacts such as those caused by body movements or device disconnection:

$$\max\left(\left|x´\left(n\right)\right|\right)>\mu \left(x´\left(n\right)\right)+5\sigma \left(x´\left(n\right)\right)$$ (1)

where *x*(n) is the filtered PPG signal, *x´*(n) is its first derivative, σ is the standard deviations (STD) and μ the mean. The second criterion ensured pulsatile amplitude was stable throughout each segment:

$$\begin{array}{c} \left(\max\left(x_{i}\right)-\min\left(x_{i}\right)\right)>1.5\;\left(\max\left(x_{j}\right)-\min\left(x_{j}\right)\right)\\ for\;i,j\in \left\{1,2,3\right\}\;and\;i\neq j \end{array}$$ (2)

where $x_{1},x_{2}\;and\;x_{3}$ are three equally sized subdivisions of *x* $\left(n\right)$ . It was not applicable to PPG-BP because of the shorter segment duration. The last criterion removed segments with extreme heart rate or with characteristics interfering with peak detection:

PR<40∨PR>220 (3)

where PR is the pulse rate in beat per minute (BPM) estimated as the average first derivative *x´*(*n*) peak to peak interval. To avoid false peaks, those with a prominence lower than 60% of the maximum prominence were discarded. The prominence of a peak quantifies the amplitude difference between its apex and its bases, computed by function *peak_prominences*.

Finally, to be able to compare the various time-based features, both datasets were resampled to a matching frequency of 250 Hz.

### 2.3 Fiducial points extraction

**FIGURE 1:**
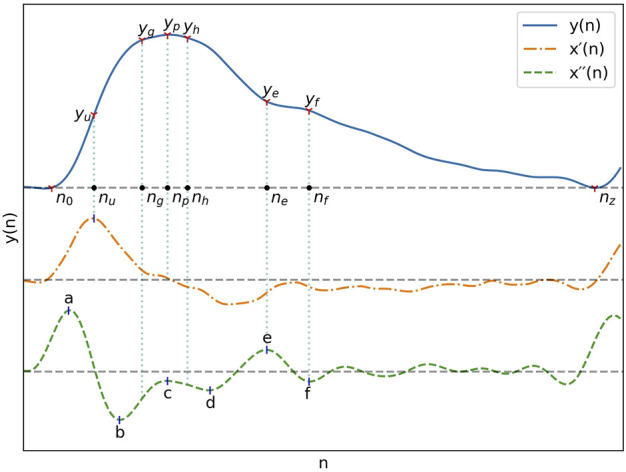
A typical PPG signal as well as its first and second derivatives with their most important fiducial points.

The fiducial points used for feature extraction are shown in Figure 1. The second derivative of the signal was first computed and low-pass filtered with a 12 Hz zero-phase sixth order Butterworth filter to obtain *x´´*(*n*), after which the third derivative *x´´´*(*n*) was computed. The PPG pulses peak positions *n*_*p*_, and their maximum upslope positions *n*_*u*_ were then established by finding the peak positions of *x*(*n*) and *x´*(*n*) with the *find_peaks* function, considering only peaks with a prominence greater than 60% of the maximum prominence. Boundaries for each pulse were established by finding the pulse onset, *n*_*0*_, associated with each *n*_*u*_. The position of *n*_*0*_ was chosen as the first positive peak of *x´´´*(*n*) left of *n*_*u*_, subject to $x´´´\left(n\right)>\;0.4\;\max\left(x´´´\left(n\right)\right)$ to ignore minor peaks. If a positive zero crossing of *x´*(*n*) could be found between that point and *n*_*u*_, it was used instead. This strategy allowed a robust detection of onset even for pulses preceded with a slow rise before the onset. The end of the pulse, *n*_*z*_, was defined as the next pulse onset. Pulses with a marked difference between the amplitude at onset and end point, satisfying $\left|x\left(n_{0}\right)-x\left(n_{Z}\right)\right|>0.12\;\left(x\left(n_{P}\right)-x\left(n_{0}\right)\right)$ , were discarded.

With pulse boundaries and peaks established, the remaining fiducial points were extracted from *x´´*(*n*). Five of those points are the *a, b, c, d* and *e* points described by Takazawa et al. (1998). Since the *e* point also marks the position of the dicrotic notch, the same nomenclature was kept for the additionnal *f*, *g* and *h* points designating the second derivative estimates of the diastolic peak, early systolic peak and late systolic peak positions. The fiducial points as described by Takazawa asusme an ideal PPG signal with well defined successive waves in the second derivative. To enable extraction from the non-ideal waveforms present in the datasets, the following five step process was developed:

1. Set the position of *a*, *n*_*a*_, to the point where *x´´*(*n*) is at its maximum and the position of *b*, *n*_*b*_, where it is at its minimum, subject to ${n<n}_{P}$ .
2. Set the position of the dicrotic notch *e*, *n*_*e*_ as the earliest *x´´*(*n*) peak with *n* constrained by $n_{P}<n<\frac{2}{3}\left(n_{z}-n_{0}\right)\wedge x\left(n\right)<0.7\;x\left(n_{P}\right)\wedge x´´\left(n\right)>\;0.05\;x´´\left(n_{a}\right)$ .
3. Set the position of the diastolic peak *f*, *n*_*f*_, as the earliest downward peak satisfying the condition $n_{e}<n<\frac{2}{3}\left(n_{z}-n_{0}\right)\wedge x´´\left(n\right)<\;0$ .
4. Set the position of *c* and *d*, *n*_*c*_ and *n*_*d*_, as the *x´´*(*n*) upward and downward peaks with the greatest difference between them, constrained by $n_{b}<n<n_{e}$ . For pulses where those peaks did not exist, the positions were estimated as the position of the maximum inflection points of *x´´*(*n*)*,* that is the maximum downward and upward peaks of the fourth derivative constrained by $n_{b}<n<n_{e}$ .
5. Estimate the position of the early and late systolic peak by setting $n_{g}=n_{b}+\frac{{n_{c}-n}_{b}}{2}$ and $n_{h}=n_{c}+\frac{{n_{d}-n}_{c}}{2}$ .

All peaks of *x´´*(*n*) and *x´´´*(*n*) were extracted by detection of the zero-crossings of the next higher order derivative.

### 2.4 Features extraction

All features were extracted on a pulse-by-pulse basis. The trend of the signal of each pulse was first removed by subtracting the linear slope connecting the start point of each pulse to its end point, as described in (Xing et al., 2020). Thus, all pulses in the resulting detrended signal, $y\left(n\right)$ , have value of zero at their starting and ending point. The amplitudes of the detrended signal at various fiducial point are hereafter designated by the form $y_{i}$ where $y_{i}=y\left(n_{i}\right)$ . The features used in this paper are recapitulated in Table 1.

**TABLE 1:** Summary of the features used in this paper.

Feature	Name/Description	Defined in
RI	Reflection index	Sec. 2.4.1, Eq. 4
AI	Augmentation index	Sec. 2.4.1, Eq. 5
$AI_{gh}$	Augmentation index of early to late systolic peaks	Sec. 2.4.1, Eq. 6
$AI_{gf}$	Augmentation index of early systolic to diastolic peaks	Sec. 2.4.1, Eq. 7
$Y_{gh}$	Amplitude ratio or early to late systolic peaks	Sec. 2.4.1, Eq. 8
IPA	Inflection point area ratio	Sec. 2.4.2, Eq. 9
$\Delta n_{ij}$	Time span between two fiducial points	Sec. 2.4.3, Eq. 10
HR	Heart rate	Sec. 2.4.3, Eq. 11
$N_{i}$	Time ratio between the portion of the pulse duration before and after a fiducial point	Sec. 2.4.4, Eq. 12
AX	Aging index	Sec. 2.4.5, Eq. 13
$S_{pe}$	Slope between points *p* and *e*	Sec. 2.4.6, Eq. 14
$S_{pf}$	Slope between points *p* and *f*	Sec. 2.4.6, Eq. 15
$W_{xx}$	Width of the pulse at *xx*% of its amplitude	Sec. 2.4.7

#### 2.4.1 Amplitude ratios

The reflection index (RI) along with the augmentation index (AI) measure the contribution of the peripheral wave reflections to the overall pulse (Elgendi, 2012). As a measure of reflected waves, AI can also be computed in regards to the early and late systolic peaks as in Eq. 6 and Eq. 7 while *Y*_*gh*_ defined in Eq. 8 is an estimate of amplitude ratio of the late to early systolic peak, which has been correlated with changes in systolic pressure (Baruch et al., 2011).

$$RI=\frac{y_{f}}{y_{p}}$$ (4)

$$AI=\frac{y_{p}-y_{f}}{y_{p}}=1-RI$$ (5)

$$AI_{gh}=\frac{y_{g}-y_{h}}{y_{g}}$$ (6)

$$AI_{gf}=\frac{y_{g}-y_{f}}{y_{g}}$$ (7)

$$Y_{gh}=\frac{y_{h}}{y_{g}}$$ (8)

#### 2.4.2 Area ratios

The Inflection Point Area ratio (IPA), the ratio of area under the curve until the dicrotic notch to the area under the curve after it, is an indicator of total peripheral resistance (Elgendi, 2012).

$$IPA=\frac{\overset{n_{e}}{\underset{{n=n}_{0}}{\sum }}y\left(n\right)}{\sum_{n=n_{e}}^{n_{z}}y\left(n\right)}$$ (9)

#### 2.4.3 Time spans

**FIGURE 2:**
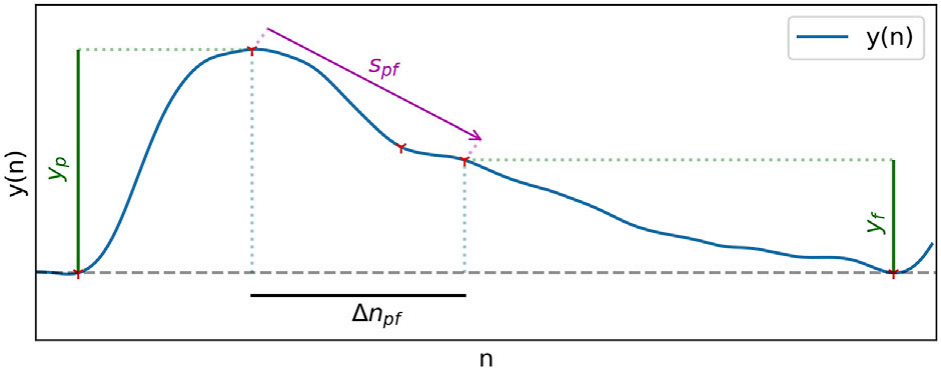
Different types of measurements used in feature extraction.

Time spans all take the same general form, given in Eq. 10, and can be visualised in Figure 2. The duration of the systolic phase, *Δn*_*0p*_, has been associated with hypertension (Dillon and Hertzman, 1941; Elgendi, 2012) while the duration of the diastolic phase, *Δn*_*pz*_, has been associated with DBP (Yoon et al., 2009). The time spans *Δn*_*0g*_, *Δn*_*0h*_, *Δn*_*gh*_, and *Δn*_*gf*_, are spans between reflected waves components, of which *Δn*_*gf*_ has been associated with pulse pressure (PP) (Baruch et al., 2011). *Δn*_*pf*_ is the time between the peak and the diastolic peak. *Δn*_*up,*_ *Δn*_*ue,*_ *Δn*_*uf*_ are time spans in relation to the maximum upslope point, of which the last has been shown to have a strong correlation with SBP and DBP (Kim et al., 2008).

$$\Delta n_{ij}=n_{j}-n_{i}$$ (10)

The HR estimation used as a feature is also, in essence, a time span, and was calculated based on the pulse duration as shown in Eq. 11, where *f*_*s*_ is the sampling rate.

$$HR=\frac{60f_{s}}{n_{z}-n_{0}}$$ (11)

#### 2.4.4 Time ratios

Three different time ratios have been included in this study, each representing the pulse duration ratio before and after a fiducial point, taking the form shown in Eq. 12. Included are *N*_*p*_ in relation to the peak, *N*_*e*_ in relation to the *e* point, and *N*_*f*_ in relation to the *f* point. The time ratio of systole to diastole, *N*_*f,*_ was shown to be correlated to SBP (Li et al., 2014).

$$N_{i}=\frac{\Delta n_{oi}}{\Delta n_{iz}}$$ (12)

#### 2.4.5 Acceleration PPG

Acceleration PPG, or second derivative PPG, is a group of features extracted from the fiducial points in the second derivative of the signal. They have been associated with arterial stiffness and vascular aging (Takazawa et al., 1998). The features *b/a*, *c/a*, *d/a* and *e/a* are amplitude ratios of the second derivative at those fiducial points, while the aging index (AX) is shown in Eq. 13.

$$AX=\frac{b-c-d-e}{a}$$ (13)

#### 2.4.6 Slopes

The slopes from the peak to the dicrotic notch, *S*_*pe*_ *,* and to the diastolic peak, *S*_*pf*_ *,* have been investigated as BP predictors. *S*_*pe*_ was shown, although with low certainty, to have a weak correlation to DBP (Kim et al., 2008), and has also been associated with peripheral resistance (Lin et al., 2020). Slopes used in this study are normalized, as in (Kim et al., 2008), in relation to the pulse peak value.

$$S_{pe}=\frac{y_{e}-y_{P}}{y_{P}\;\Delta n_{pe}}$$ (14)

$$S_{pf}=\frac{y_{f}-y_{P}}{y_{P}\;\Delta n_{pf}}$$ (15)

#### 2.4.7 Widths

Widths are conceptually the same as time spans, but they are not calculated from specific fiducial points in the pulse. Rather, the span is the width of the pulse at a certain percentage of its amplitude. It has been used as a BP predictor (Ding et al., 2019) and is associated with systemic vascular resistance (Awad et al., 2007). In this study, the pulse width is measured at 30%, 50%, 70% and 90% of $y_{p}$ for $W_{30},W_{50,}W_{70}$ and $W_{90},$ respectively.

#### 2.4.8 Outlier exclusion and feature vector construction

Outlier exclusion was performed on a per-pulse basis. Morphologically abnormal values for IPA were identified first and any values below 0.5, usually caused by an abnormal shape of the diastolic part of the pulse, were rejected. The feature vectors of both datasets were then temporarily joined to compute the global mean and the global STD, $\sigma_{global},$ of each feature. Pulses where any feature diverged more than 4 STD from the mean were considered outliers and rejected.

The remaining feature vectors for pulses in the same segment were then averaged and saved. Since only a single BP measurement is provided per subject in the PPG-BP dataset, features extracted from different segments but from the same subject were also averaged together.

### 2.5 Statistical comparison of the datasets

To characterise the differences between PPG-BP and UCI, the distribution of features and BP data compiled in section 2.4.8 were first examined.

For each feature, as well as SBP and DBP a two sample Kolmogorov–Smirnov (KS) test was performed with α = 0.001 to determine if differences between distributions were significant.

For each dataset, the mean and STD of each feature was calculated. For each feature, the difference between the mean value of the two datasets, was determined as per Eq. 16. The same was also done for the STD value as in Eq. 17. The results were computed as a percentage of $\sigma_{global}$ to bring them on a comparable scale. This analysis was also done on SBP and DBP.

$$\mu_{\%}=\frac{\mu_{uci}-\mu_{ppg-bp}}{\left|\sigma_{global}\right|}\cdot 100$$ (16)

$$\sigma_{\%}=\frac{\sigma_{uci}-\sigma_{ppg-bp}}{\left|\sigma_{global}\right|}\cdot 100$$ (17)

Since many features are affected by the pulse duration, those tests were then repeated with HR compensation. That is to say that all time spans (Section 2.4.3) and widths (Section 2.4.7) were multiplied by HR while slopes (Section 2.4.6) were divided by HR before recomputing $\sigma_{global}$ , Eq. 16 and Eq. 17, yielding $\mu_{adj\%}$ and $\sigma_{adj\%}$ .

Finally, the feature correlation matrix was computed: for each feature, the Pearson correlation coefficient (*r*) was calculated against every other feature. The difference between the correlation matrix of each dataset was then produced to highlight their discrepancies.

### 2.6 Response to BP variations and shared predictive power

#### 2.6.1 BP correlation test

The Spearman rank correlation coefficient ( ρ) was computed to assess correlation between each feature and SBP as well as DBP, respectively. Spearman correlation was selected here instead of Pearson for its ability to identify both linear and non-linear relationships. The difference between the datasets was then computed to reveal any divergence in BP-features relationship.

#### 2.6.2 BP estimation test

For this section, the *Scikit-Learn* machine learning library was used (Pedregosa et al., 2011). Using the *svm.svr* module, a support vector regression (SVR) model with a radial basis function (RBF) kernel was trained for SBP estimation on the PPG-BP dataset and another on UCI, keeping one random sample per subject. Therefore, when splitting a dataset into a training and testing set, data from one subject was never included into both the training and testing set.

To counter the bias caused by the non-uniform sample distribution, sample weights were passed to the model for training and also in subsequent evaluation of performance. Samples were first split into 12 equally spaced bins based on their BP value. The weight *g* of each sample was $g_{i}=k_{\max}/k_{i}$ where $k_{\max}$ is the number of samples in the bin with the most samples and $k_{i}$ is the number of samples in the current sample’s bin. Because samples were concentrated in the middle of the BP range, the resulting weights increased emphasis on the samples towards the extrema of the BP range, as to approximate training and testing using a uniform distribution.

The features were centered to zero mean and scaled to unit variance before being handed to the model.

The model regularization parameter *C*, controling penalization of estimation errors during training, and the kernel function scale parameter γ , were optimized first through a coarse then a fine parameter grid search, as described in (Hsu et al., 2016). A leave-one-out cross-validation strategy was used to maximize the ammount of useable data for training.

Backward feature elimination was used to find the optimal feature set for each dataset, following this method:

1. Using 10-fold cross validation, sequentially train and test the SVR using all features but one, until all features have been left out once.
2. Compare the results and save the reduced feature set with the best cross-validated performance.
3. Restart from step one using the reduced feature set until only 4 features remain.
4. Select the optimal feature set, that is the one that had the best performance throughout the entire process.

At every step, performance was evaluated using the weighted coefficient of determination R^2^, as defined in Eq. 18, where *i* is the sample index, $g_{i}$ is the sample weight, $u_{i}$ is a sample’s true BP, ${\hat{u}}_{i}$ is a sample’s estimated BP, $\bar{u}$ is the weighted mean of the true BP of all samples defined in Eq. 19, and k is the number of samples.

$$R^{2}=1-\frac{\overset{k}{\underset{i=1}{\sum }}g_{i}{\left(u_{i}-\hat{u_{i}}\right)}^{2}}{\sum_{i=1}^{k}g_{i}{\left(u_{i}-\bar{u}\right)}^{2}}$$ (18)

$$\bar{u}=\frac{\sum_{i=1}^{k}g_{i}u_{i}}{\sum_{i=1}^{k}g_{i}}$$ (19)

The Pearson correlation coefficient between the estimated BP and true BP, as well as the MAE of the estimated BP were used as secondary metrics. In addition to plotting the estimated BP and true BP pairs for each test, Bland-Altman plots (Bland and Altman, 1986) were also produced to allow better interpretation of the results. Final performance evalution with the optimised model parameters was carried out through leave-one-out cross-validation on the training dataset. The models were then retrained separately on their entire respective training dataset without leaving out any samples, but keeping the same set of features as well as the same *C* and γ values. Those retrained models were then validated against the other dataset to see if the predictive power would be retained.

## 3 Results

### 3.1 Pre-processing and feature extraction

For PPG-BP, 16 of the dataset’s 657 segments were rejected by criterion 1) before feature extraction. No segments were rejected due to criterion 2) or 3). From the remaining segments, 742 pulses were identified, of which 22 (3%) were rejected as outliers based on extracted feature values. Averaging the remaining pulse features per segment yeilded 533 valid segments with complete feature vectors, for an overall segment rejection rate of 19%. After averaging per subject, the dataset had 211 feature vectors.

For UCI, 2,376 records were too short to generate the segments and were ignored. The remaining records yielded 48,120 segments, of which 1791 were rejected due to non-pulsatile ABP signals, 1,228 because criterion 1), 2,663 because of criterion 2) and 78 because of criterion 3). From the remaining segments, 83,903 pulses were identified, of which 7,104 (8%) were rejected as outliers based on extracted feature values. Averaging the valid pulses per segment yeilded 21,698 valid segments with complete feature vectors, for an overall segment rejection rate of 55%.

### 3.2 Statistical comparison

**FIGURE 3:**
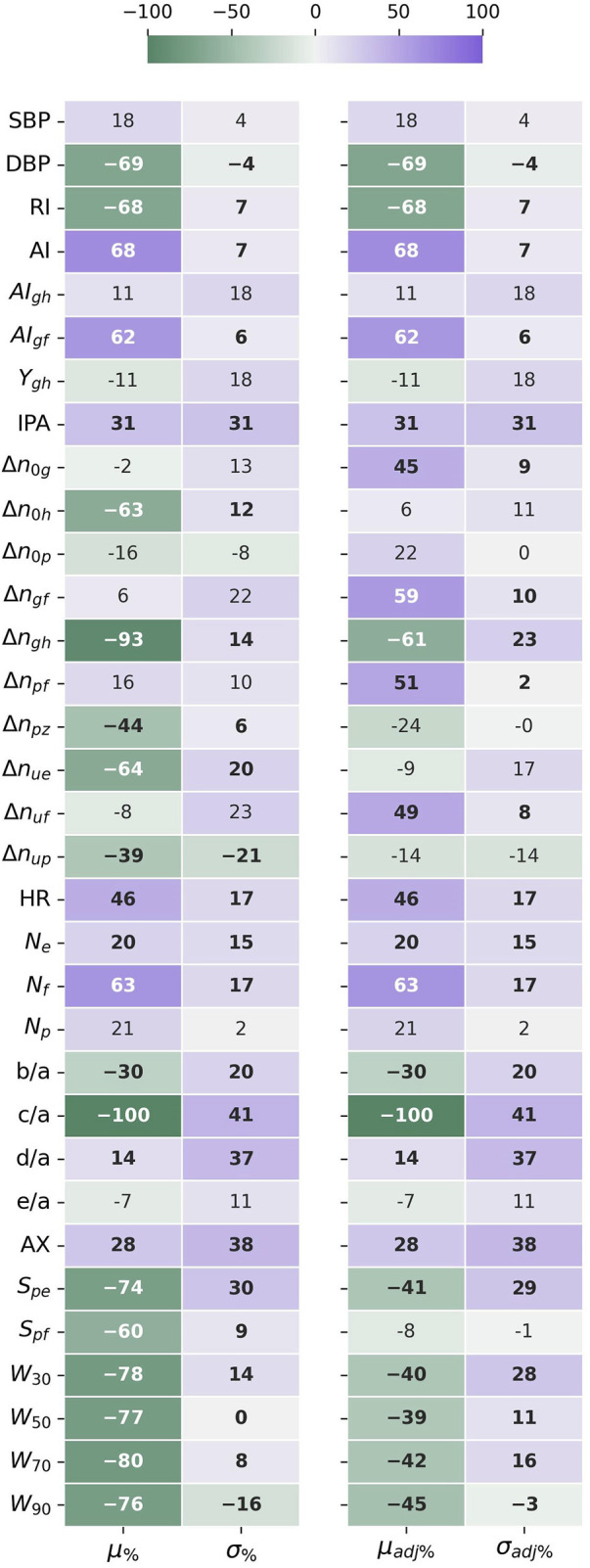
The difference in mean (μ%) and standard deviation (σ%) between the datasets, given as a percentage standard deviation of the joined datasets. The HR adjusted forms (μadj%) and (σadj%), compensate for the different HR distributions affecting time sensitive features. Negative values indicate that the mean or std values for UCI are lower than for PPG-BP. Values in bold indicate significantly different distributions (p < 0.001) according to the Kolmogorov-Smirnov test.

Results of the statistical comparison of the datasets are aggregated in Figure 3.

According to the KS-test, the differences between feature distributions were significant $\left(p<0.001\right)$ for 22 out of 31 features for the original features and 21 out of 31 features for the HR adjusted features.

**FIGURE 4:**
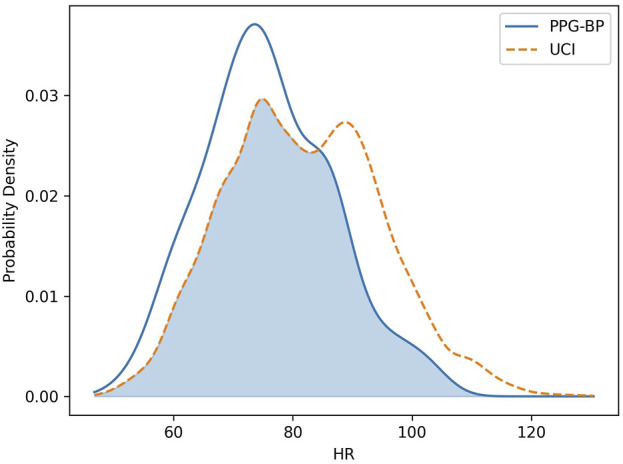
Comparison of the HR distribution for the PPG-BP and UCI datasets.

Looking at $\mu_{\%}$ the difference in mean original feature values between the datasets, *c/a* stood out among all features, registering a difference of −100% of the standard deviation on UCI compared to PPG-BP. Several other features displayed a large difference, the second highest being $\Delta n_{gh}$ (−93%), followed by the width features all showing at least −75% difference, *S*_*pe*_ (−74%), AI (68%) and RI (−68%). The difference between HR distributions (46%) is worth noting because of its direct physiological implication and its effect on other features. As shown in Figure 4, the UCI HR distribution is bimodal with a first peak positioned around 75 BPM, similar to PPG-BP, and a second peak close to 90 BPM. The average HR was 6.2 BPM higher in UCI and 28% of segments had a HR above 90 compared to 8% in PPG-BP.

Because HR directly affects the value of many features, looking at the HR corrected difference in mean $\mu_{adj\%}$ reveals what part of $\mu_{\%}$ is not explained by the difference in HR distribution, and highlights fundamental differences in the pulse shapes. Values significantly higher on UCI were AI (68%), *N*_*f*_ (68%), AI_*gf*_ (62%), $\Delta n_{gf}$ (59%), $\Delta n_{pf}$ (51%), $\Delta n_{uf}$ (49%), HR (46%), $\Delta n_{0g}$ (45%), IPA (31%), AX (28%) and *d/a* (14%). Values significantly lower on UCI were *c/a* (-100%), RI (-68%), $\Delta n_{gh}$ (−61%), *W*_*90*_ (−45%), *W*_*70*_ (−42%), *S*_*pe*_ (−41%), *W*_*30*_ (−40%), *W*_*50*_ (−39%) and *b/a* (−30%).

The five features with the highest STD difference were *c/*a (41%), AX (38%), *d/*a (37%), IPA (31%) and *S*_*pe*_ (30% or 29% adjusted for HR), all higher on UCI*.* In fact, STD was higher in UCI for 87% (or 80% adjusted for HR) of features, indicating a greater variability in pulse morphology within the dataset.

The relation of those differences to differences in pulse morphology between UCI and PPG-BP are illustrated in Figure 6. For example, the PPG-BP pulse with typical values (A) had a well defined second derivative peak for the *c* point with *c/a* = −0.15 while the depression at the *c* position for the bottom two pulses, (C) and (D), gave lower values of *c/a* = −0.40 and *c/a* = −0.68. The *f* point was also positioned later in the pulse for (C) and (D), resulting in larger time spans. Pulse (C) had *S*_*pe*_ = −0.022, $\Delta n_{0g}$ = 36; $\Delta n_{uf}$ = 83; $\Delta n_{pf}$ = 69 and *N*_*f*_ = 1.22 which can be directly compared to the values of (A), *S*_*pe*_ = −0.012, $\Delta n_{0g}$ = 32, $\Delta n_{uf}$ = 76, $\Delta n_{pf}$ = 50 and *N*_*f*_ = 0.90, since both had similar heart rates.

Pulse (C) also had a very narrow peak section with *W*_*90*_ *=* 14 while the PPG-BP pulse (A) had a wider one with *W*_*90*_ *=* 30. The heart rate of the UCI pulse (B) was 20 BPM lower than the PPG-BP pulse (A) but still only had *W*_*90*_ *=* 15. Pulse (B) also had AI = 0.76 because of the larger amplitude difference between *p* and *f* as well as a lower *b/a =* −1.26 caused by its more pronounced *b* peak in the second derivative. In comparison the PPG-BP pulse (A) had AI = 0.50 and *b/a =* −0.79. The variability of *c/*a in UCI is also illustrated in Figure 6, where the amplitude of *c* can be seen fluctuating between zero and the amplitude of *b* in the three UCI pulses. It should be noted that the pulses in Figure 6 are not archetypal pulses of UCI, which includes highly varied pulse shapes. The pulses in Figure 6 were rather selected to illustrate the morphological features that induce some of the largest feature distribution differences observed between the datasets.

**FIGURE 5:**
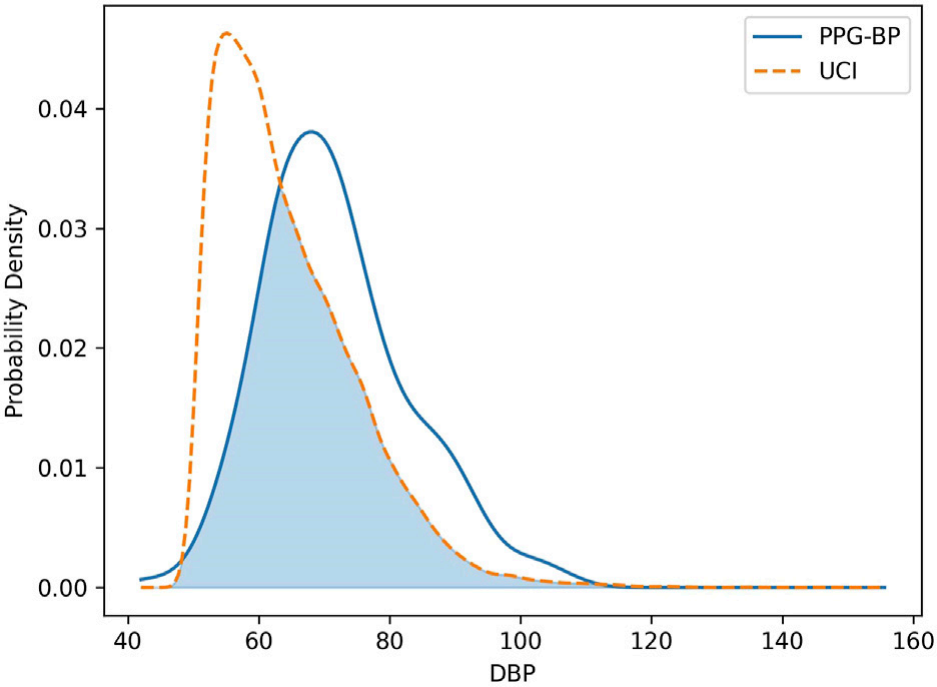
Comparison of the DBP distribution of the PPG-BP and UCI datasets.

In regards to BP, the SBP distribution was similar for both datasets and close to normality. However, the DBP distributions had significant differences. The average DBP value for UCI was lower at 64.3 mmHG, compared to 71.8 mmHG for PPG-BP, or a difference equivalent to -64% of the global sandard deviation. The DBP distribution of UCI was also found to deviate significantly from normality, as shown in Figure 5, with a slightly leptokurtic shape and a significant skew towards lower values.

Overall, the datasets had a similar degree of internal correlation, with $mean\left(\left|r\right|\right)=0.36$ compared to $mean\left(\left|r\right|\right)=0.35$ for PPG-BP. UCI had 61% of feature pairs with $\left|r\right|>0.25$ and 28% of feature pairs with $\left|r\right|>0.50$ , as compared to 57% and 25%, respectively, for PPG-BP.

As for the correlation between features, the largest differences between datasets were observed with *S*_*pe*_, a feature that also displayed a significant mean and STD differences between the datasets. Compared to PPG-BP, the correlation level *|r|* of *S*_*pe*_ increased on average by 0.40 with seven other features in UCI: *b/a*, *d/a*, *AX*, $\Delta n_{up},\Delta n_{0p},Y_{gh}\;and\;AI_{gh}$ . Another important difference was *e/a*, which had a correlation of *r = -*0.42 with *d/a* for PPG-BP, while that correlation fell to *r =* 0.02 for UCI.

**FIGURE 6:**
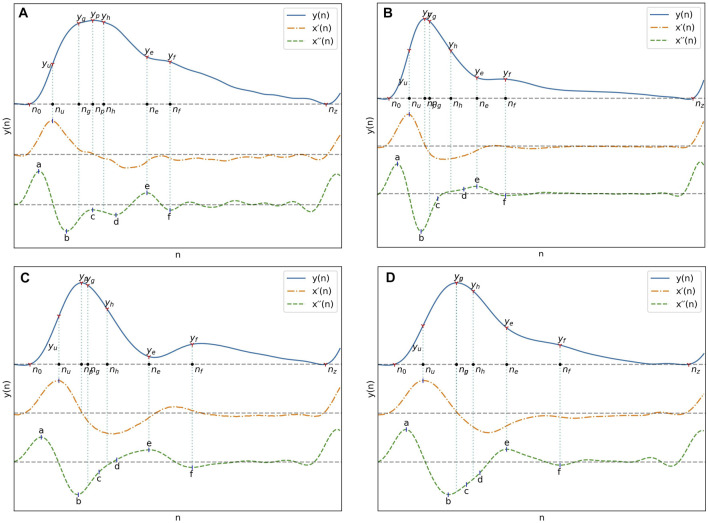
**(A)** Pulse from PPG-BP with characteristics representative of the dataset. **(B)**, **(C)** and **(D)** Pulses from UCI illustrating some of the differences observed with PPG-BP. In general, the pulse shape was more pointed and narrower, dropping sharply after the peak. The amplitude of the PPG signal was usually lower at the e and f points, and the f point was often encountered later in the pulse. The second derivative showed a lot of variability, but compared to PPG-BP, the b point had usually a lower amplitude and the c and d points were often not well-defined peaks in the second derivative and were thus estimated from the inflection points. This resulted in highly variable but general lower amplitude values for the c point especially, compared to PPG-BP where it more consistently appears as a peak with a value closer to zero. Note that the pulse duration is normalized in all four pulses of this figure.

The differences observed between the datasets were in large part associated with the presence of particularily pointed pulses in UCI and rare in PPG-BP. Those pulses hold a different relationship between features compared not only to most pulses in PPG-BP, but also to other types of pulses in UCI, increasing variability. Their caracteristics can be seen in the UCI Pulses of Figure 6. In general their *c* and *d* points were not well defined peaks in the second derivative, but inflection points in a curve between *b* and *e*. The amplitude of *d* tended to be higher as *e* also got higher and the *S*_*pe*_ slope became more pronounced. *AX,* which is calculated from the amplitude of the second derivative fiducial points, was in turn affected. Those pulses were also associated with a quick pulse onset with shorter $\Delta n_{up},\Delta n_{0p}$ and a proportionnaly narrower pulse wave. Finally the *g* point also tended to be situated around the peak while the *h* point came later at a much decreased PPG amplitude, wherea in PPG-BP the amplitude at *g* and *h* was not related to *S*_*pe*_ due to their more varied positions around a generally flatter pulse peak.

### 3.3 Response to BP variations and shared predictive power

#### 3.3.1 Correlation to BP

**FIGURE 7:**
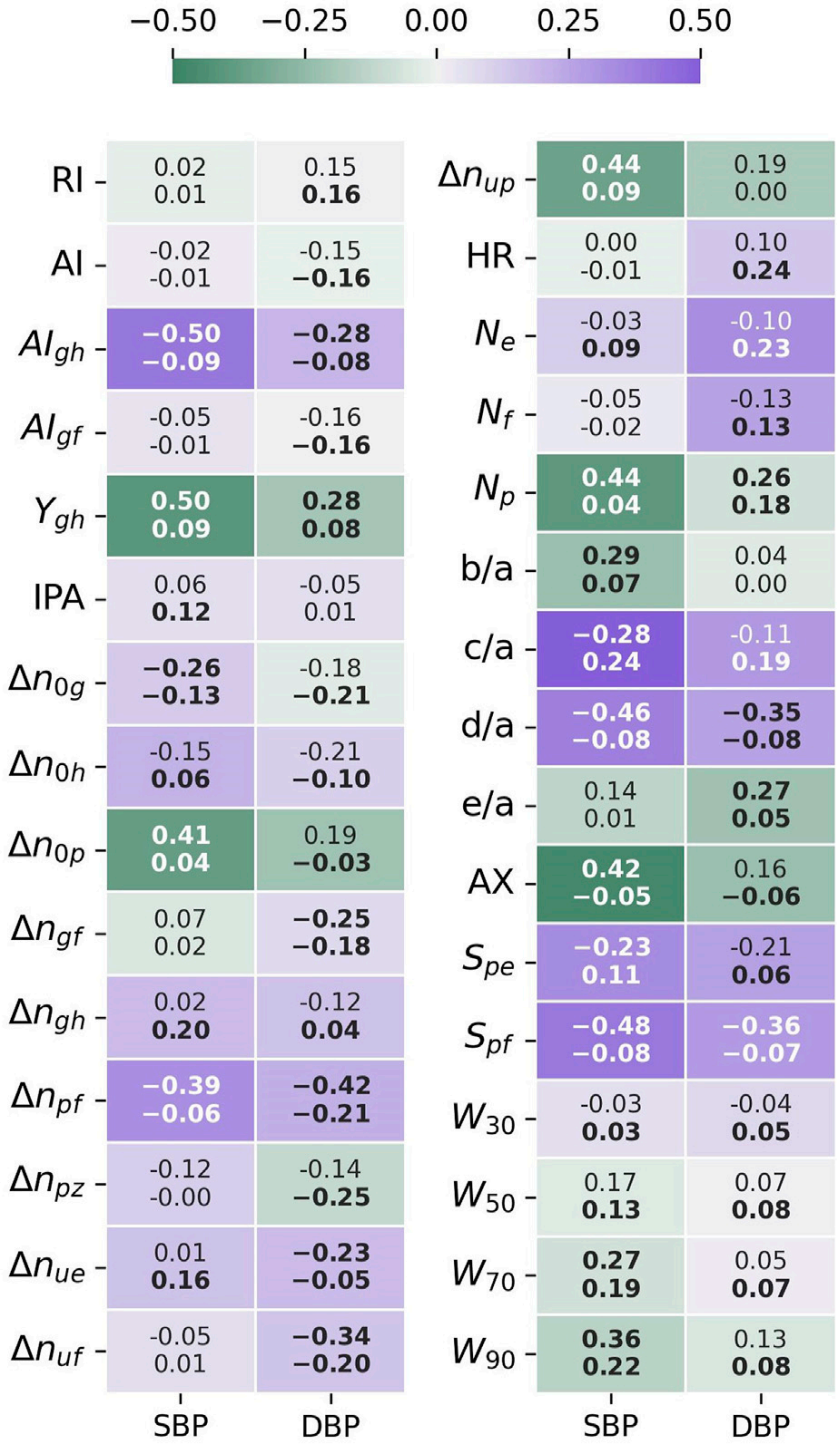
Feature-BP Spearman correlation test results. The top value is the correlation coefficient for PPG-BP, the bottom value the correlation coefficient for UCI, while the color and intensity show by how much UCI differs from PPG-BP. Values in bold indicate that the correlation is significant (*p* < 0.001).

The Spearman rank correlation coefficient ( ρ) of each dataset’s features against SBP and DBP is presented in Figure 7.

For SBP, significant correlation could be established for 15 features in PPG-BP. The three most correlating features were $AI_{gh}\left(\rho =-0.50\right),$ $Y_{gh}\left(\rho =0.50\right),$ $S_{pf}\left(\rho =-0.48\right)$ , and a total of 14 features had a correlation of $\left|\rho \right|>0.25$ . For UCI, the three most correlating features were c/a (ρ=0.24 ), $W_{90}\;(\rho =0.22$ ) and $\Delta n_{gh}\;(\rho =0.20$ ). It should be noted that those features all had major mean and STD differences with PPG-BP (see Section 3.2). In total, significant correlations with SBP could be established for 22 features in UCI, although coefficients were lower at $\left|\rho \right|\leq 0.25$ for all features.

Stronger correlation with SBP in one dataset was not associated to a stronger correlation with SBP in the other dataset. For example, the three most correlating features of PPG-BP, $AI_{gh},$ $Y_{gh}\;and$ $S_{pf}$ ( $\left|\rho \right|\geq 0.48$ ) were not among the higest in UCI, where their SBP correlation reached at most $\left|\rho \right|=0.09$ . As for the most correlating features in UCI, $W_{90}$ obtained ρ=0.36 in PPG-BP, $\Delta n_{gh}$ was not significant and *c/a* had a stronger but opposite correlation of ρ=−0.28 . Two other features showed significant but reversed correlation, although to a lesser degree: $S_{pe}$ with ρ=−0.23 for PPG-BP and ρ=0.11 for UCI, and AX with ρ=0.42 for PPG-BP and ρ=−0.05 in UCI.

A similar pattern was observed for DBP. Significant correlations were established for ten features for PPG-BP. Those with the highest correlation were ${\Delta n}_{pf}\left(\rho =-0.42\right)$ , $S_{pf}\left(\rho =-0.36\right),and\;d/a$ $\left(\rho =-0.35\right)$ . For UCI, significant correlations were established for a total of 28 features. Those with the highest correlation were ${\Delta n}_{pz}\left(\rho =-0.25\right),$ $HR\left(\rho =0.24\right),$ and $N_{e}\left(\rho =0.23\right)$ . In addition, relatively strong correlation (for UCI) was shared with one of the most correlating features of PPG-BP: ${\Delta n}_{pf}\left(\rho =-0.21\right)$ . Again for DBP, correlation levels of $\left|\rho \right|>0.25$ were only reached on PPG-BP, and that for nine of the ten features where significance was attained.

#### 3.3.2 BP estimation

Sampling one feature vector per subject in UCI for the BP estimation test yielded a total of 7,087 vectors. Parameter selection for the PPG-BP trained model resulted in C=75,γ=0.1 while selected parameters for UCI were C=0.25,γ=0.03 . For PPG-BP, eight features were retained during feature selection: $N_{e},S_{pf},W_{90},\Delta n_{gf},\Delta n_{gh},\Delta n_{pf},AX\;and\;HR$ . For UCI, sixteen features were retained: $AI_{gf},N_{f},S_{pf},W_{30},W_{50},W_{90},\Delta n_{0g},\Delta n_{0h},\Delta n_{uf},\Delta n_{gh},HR,AX,RI,b/a,c/a$ and d/a .

**FIGURE 8:**
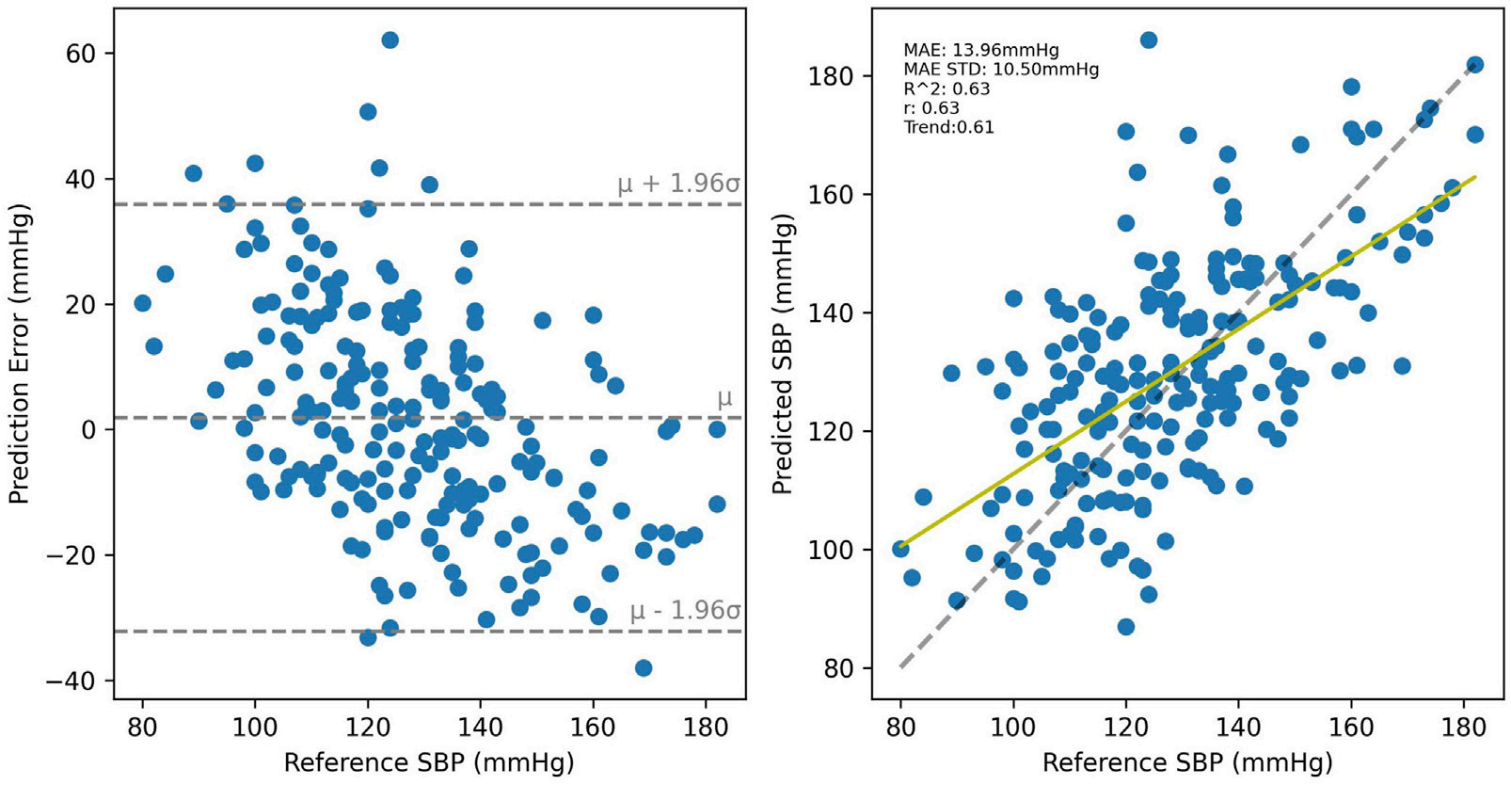
Cross validated results for the PPG-BP trained model.

**FIGURE 9:**
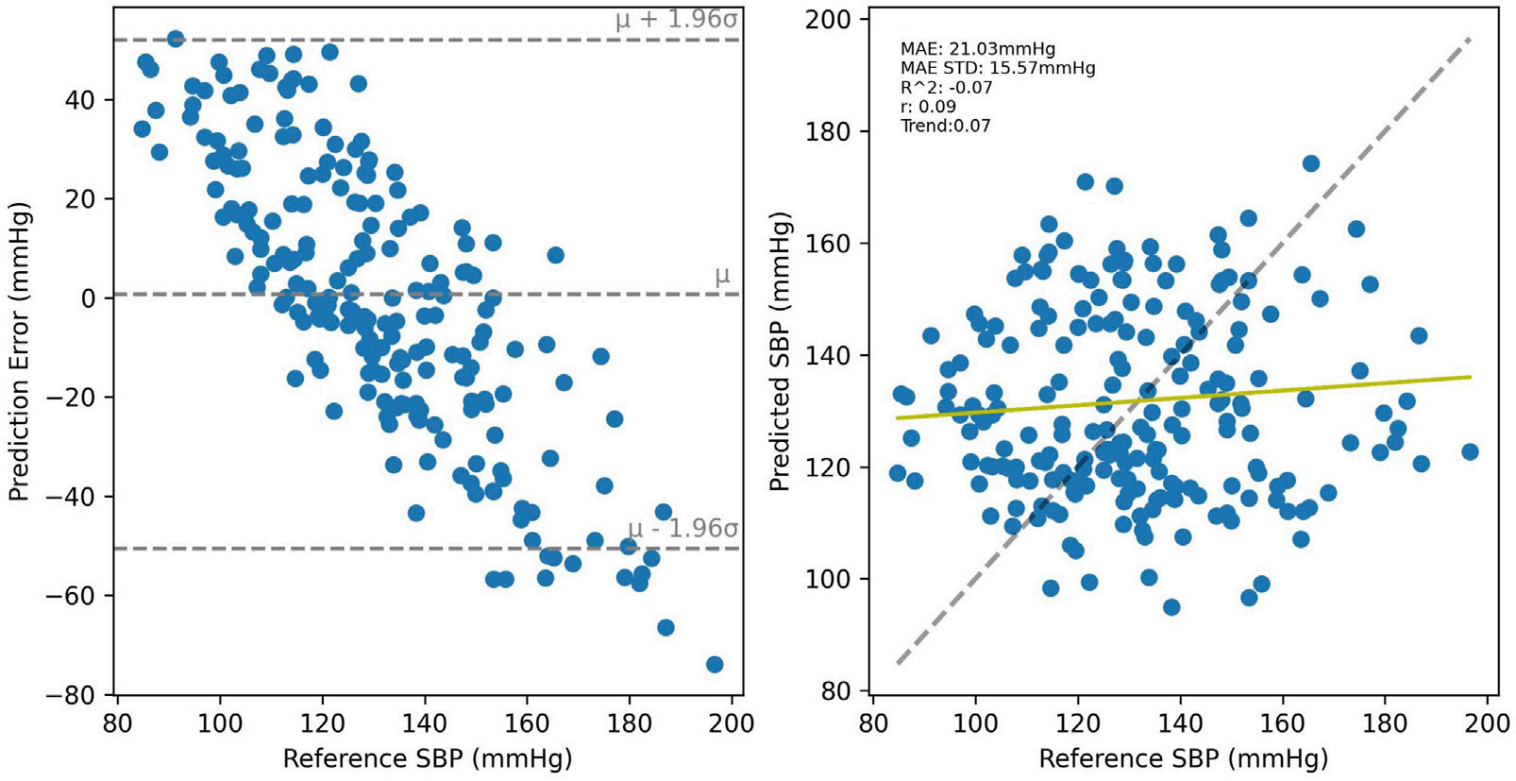
Results of the PPG-BP trained model tested with the UCI data. A random sample of the BP estimations are shown but the metrics are for the entire dataset.

SBP estimation results for the PPG-BP trained model are presented in Figure 8 for cross-validated tests on PPG-BP. During cross-validated tests, the model tended to overestimate samples with low BP values and underestimated samples with high BP. Nonetheless, it showed significant predictive power over the entire range of BP values, as shown by the R^2^ score of 0.63. Secondary metrics were *r =* 0.63 and MAE = 13.96 mmHg with an STD of 10.50 mmHg. When applied to predict SBP for the UCI dataset, a model with the same parameters trained with the entire PPG-BP did not retain any predictive power, as shown in Figure 9, giving worse results than a mean predictor, as shown by the R^2^ score of -0.07. Secondary metrics were *r* = 0.09 and MAE = 21.03 mmHg with an STD of 16.95 mmHg.

**FIGURE 10:**
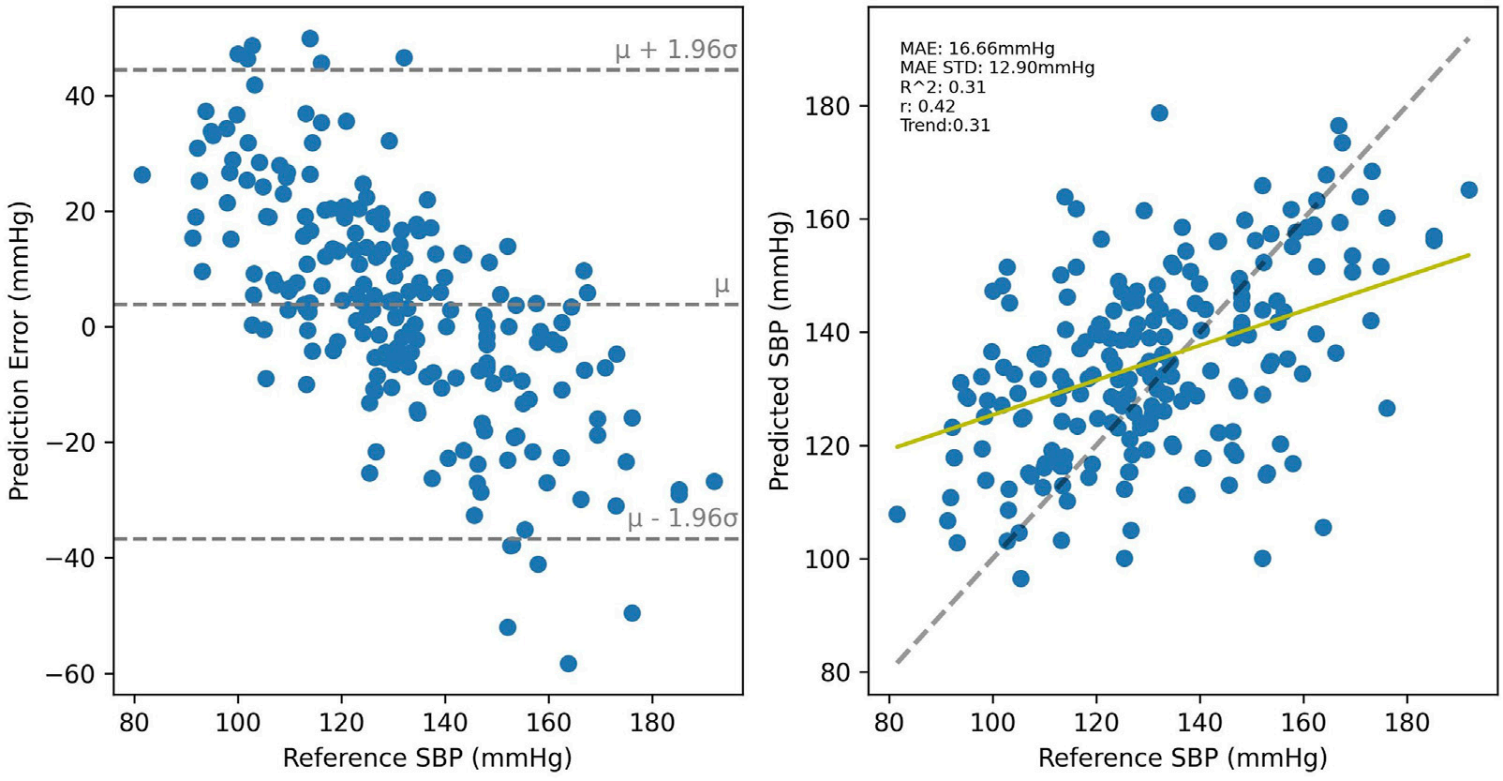
Cross validated results for the UCI trained model. A random sample of the BP estimations are shown but the metrics are for the entire dataset.

**FIGURE 11:**
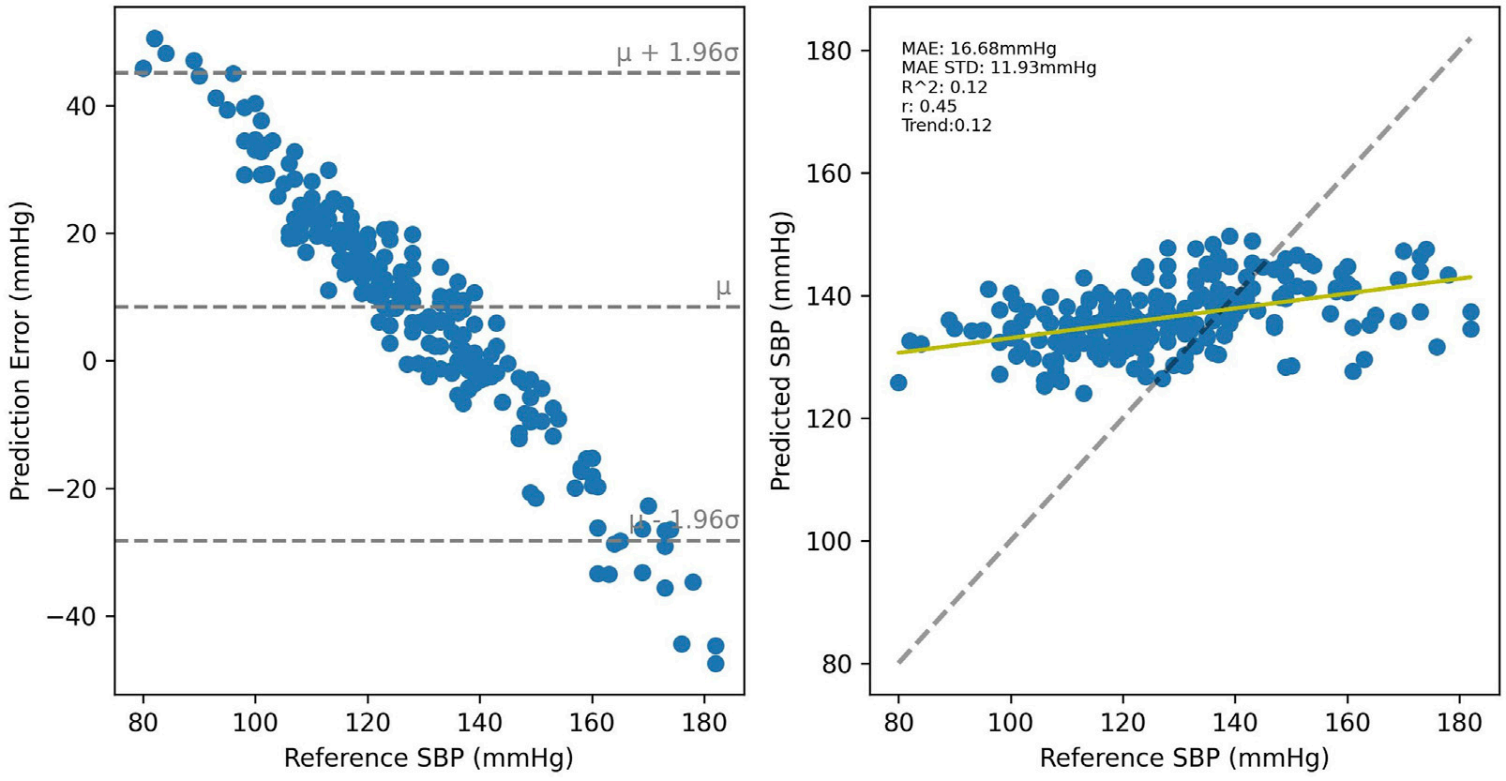
Results of the UCI trained model tested with the PPG-BP data.

Cross-validated results for UCI, shown in Figure 10 were considerably worse than for PPG-BP, achieving only R^2^ = 0.31, with secondary metrics *r =* 0.42 and MAE = 16.66 mmHg with an STD of 12.90 mmHg. Applying the UCI trained model to PPG-BP resulted again in a loss of predictive power, as shown in Figure 11, although not as dramatic as for the PPG-BP trained model applied to UCI. It resulted in an R^2^ score of 0.12, barely better than a mean predictor. Secondary metrics were *r* = 0.45 and MAE = 16.68 mmHg with an STD of 11.93 mmHg. The model can in fact be said to almost act as a mean predictor, as the produced BP values always remain close to the mean BP, with an STD of 5.74 mmHg.

## 4 Discussion

Analysis of the BP and features distributions showed fundamental differences between datasets. Because vascular aging plays a role in shaping the pulse wave, it can be hypothesized that differences in the age distribution between the datasets could influence the results. This hypothesis can neither be confirmed nor rejected as age information is not available for UCI. While we can’t ascertain that the UCI data is similarily distributed, it can be interesting to look at age data available for 2040 subjects of the MIMIC waveform database that have been matched to the MIMIC clinical database. That information, provided in the MAP-CW file of the dataset, shows an average age of 65 ± 17. It should however be considered a low estimate since age for patients older than 90 years of age is simply noted as “90+”.

For several features, the difference can be partly attributed to the higher mean HR in UCI which results in narrower pulses. That characteristic of UCI and more specifically the large portion of data associated with a HR above 90, could be linked with stress or poor health. It supports the idea that differences in the conditions in which the data was obtained, or in the condition of the subject, has an influence on the data.

However, even when scaled by the heart rate, the difference in mean values between the datasets remained significant. That remainder was linked to morphological variations between the datasets, notably to the UCI pulse types illustrated in Figure 6. Those have a more pointed peak, more of their energy concentrated early in their period, and often lack well defined *c* and *d* peaks in the second derivative.

The loss of correlation between *d/a* and *e/a* on UCI may give some insight into the physiological origin behind the differences. The *e/a* ratio is associated with an increased inflection at the dicrotic notch while the *d/a* ratio is associated with inflection at the late systolic peak. A lower *d/a* ratio often equates to a flatter PPG peak with a sharp drop, as compared to a pointier PPG peak with a more progressive decline for higher ratios. In the wave-reflection based PPG model, this can be seen as the effect of timings and amplitude between the main systolic peak and the renal and iliac reflections (Baruch et al., 2011). The correlation between those features in the PPG-BP indicates that relatively uniform parameters in the circulatory system of the subjects define both reflections, while the loss of correlation on UCI indicates less uniformity, since the renal and iliac reflections appear modulated by different parameters. The *d/a* and *e/a* ratios have been shown to change independently with the administration of vasodilators or vasoconstrictors (Takazawa et al., 1998), which hints at possible differences in subject or environmental conditions between UCI and PPG-BP.

The relatively high degree of linear correlation between features was expected, as many features are similar in nature and are influenced in the same way by the pulse characteristics. For example widths and timings are all expected, to a certain degree, to vary together with the pulse duration.

The BP correlation test showed a different relationship between features and BP for each dataset. The higher correlation coefficients generaly found on PPG-BP indicated a more uniform response between the subjects, which is coherent with the controlled data collection and subject selection methodology of PPG-BP, whereas the data from UCI lacks any control over environmental and subject conditions. For UCI, two of the features correlating the most with SBP were *c/a* and $\Delta n_{gh}$ , features that also had the most difference between the datasets. Since those features had an opposite or null correlation on PPG-BP, the difference points to possible clusters of patients or conditions in UCI where consistant BP changes accompany those morphological changes. In fact, 35% of the pulses had a *c* amplitude lower than *d* in UCI, while it is the case for only 5% of pulses in PPG-BP. In UCI, those pulses were associated with an average SBP lower by 10.7 mmHg and average DBP lower by 3.8 mmHg compared to pulses with well defined second derivative peaks where *c > d*.

Of the features retained by the SBP estimation model for PPG-BP, four out of eight ( $S_{pf},W_{90},\Delta n_{pf}$ and AX) had significant correlation to SBP. Some of the feature that showed among the strongest correlation were not retained, which may be attributed to information redundance due to the generally strong correlation between features. For UCI, the large sample size allowed establishing significance at lower correlation levels, despite the increased variability of the data. The three features with the highest correlation to SBP ( $W_{90},\Delta n_{gh}$ and *c/a*) were retained for estimation, the latter two also being the two features with the largest difference in mean value between datasets. Despite significant SBP correlation being present for *b/a*, *c/a* and *d/a*, no second derivative ratios were retained for PPG-BP. The fact that those three ratios were retained for UCI, and especially *c/a* with its opposite correlation profile compared to PPG-BP, maybe related to the aforementioned presence of clusters of patients with significant differences in the second derivative. It is also interesting to note that HR was retained for both datasets despite the absence of direct correlation to SBP, which suggests that scaling of some features in relation to the pulse duration played an important part in the estimation process.

Performance of BP estimation algorithms are extremely difficult to compare. The absence of a standard test dataset and the tradition of reporting the results in mmHg mean error or MAE make the results very sensitive to sample selection and BP range. Sample size, preprocessing and sampling methods vary widely, and are not always clearly described in published studies. Comparison with a few other calibration free studies can be made but should not be seen as decisive. Kachuee et al. obtained an MAE of 11.17 ± 10.09 mmHG and *r* = 0.59 on UCI using Adaboost, but also making use of ECG (Kachuee et al., 2017). Slapnicar et al. obtained an MAE of 15.41 mmHg on 510 MIMIC subjects with a deep neural network on the raw PPG signal and the two first derivatives, while 18.34 mmHG was obtained when using a random forest algorithm with hand crafted features (Slapničar et al., 2019). As a last example, Maqsood and al. tested the same algorithms on both PPG-BP and MIMIC (although without cross-dataset tests) and reported an MAE of 5.32 ± 4.26 mmHG for PPG-BP and 5.59 ± 5.92 mmHg for MIMIC with a bidirectional long short-term memory neural network (Bi-LSTM) and time domain features, while they obtained 15.48 ± 3.52 mmHG for PPG-BP and 12.14 ± 6.67 mmHG with an SVR (Maqsood et al., 2021).

While the use of more complex models such as Bi-LSTM may potentially bring uncalibrated BP estimation closer to medical device requirements, optimal performance was not the goal of this study and a simpler model was prefered to illustrate the impact of observed differences. The present results are thus more in line with those of other simpler models. More importantly, the present results clearly show that what was learned on one dataset does not apply, or applies only weakly, to the other. It is interesting to note that with fewer features, the cross-validated model of PPG-BP obtained an R^2^ twice as that of UCI. The fact that less features and thus less information is necessary to get those results in PPG-BP indicates a more uniform response in the subjects, which may be due to the more controlled data collection conditions. This would also explain why the PPG-BP trained model retains no predictive power at all with UCI, since it would not cover the wider variety of patients and recording conditions present in UCI, while the UCI trained model, having knowledge of a wider variety of conditions, may be able to retain some power, even though very weak, when applied to PPG-BP.

Absolute values of the PPG signal can vary greatly depending on the recording conditions and equipment calibration. To ensure a consistent comparison between the different records, and especially between datasets possible, no raw amplitudes were used as features, neither was the DC component of the signal. Thus, a part of the signal information, which could potentially improve performance, was not used. The added benefit of this information in the case of UCI is however doubtful, as amplitudes were uneven between segments, with the pulsatile amplitude actually following a strict bimodal distribution with a wide separating gap.

Another factor limiting the comparison was the structure of PPG-BP, which offers three short PPG segments per patient, all associated to a single BP value. In contrast, UCI offers longer segments with continous a contnuous ABP signal. The use of 5 s samples of UCI allows to obtain on average the same number of usable pulses as in three PPG-BP segments, and to reduce the ABP signal to mean SBP and DBP over the period. As a result of those differences, two additional criteria had to be applied to UCI in Section 2.2 to ensure the integrity of the signal. One on the ABP signal to detect non-pulsatile ABP, and one on the PPG signal to ensure consistant pulsatile amplitude throughout the segment. We believe that those additional criteria should not affect the validity of the comparison. They merely ensure the signals are present and usable to the same degree as in PPG-BP, which was already similarily screened for signal integrity prior to its release. However, another aspect of those differences brings uncertainty to the cross-dataset validation. While in UCI the BP measurements are derived from the ABP signal corresponding to the 5 s PPG segment, the BP measurements in PPG-BP are derived from a period of 30 s preceding the acquisition of the PPG signal. Moreover, unknown gaps exist between the three PPG segments, the only guarantee being that the BP measurements and all PPG segments are taken within a period of 3 min. While this is not an idea situation and may ultimately produce a certain degree of decoupling between the PPG signals and the recorded BP value, the simultaneous acquisition of BP and PPG may not be as important for PPG-BP as for UCI, where ABP signals sometimes change rapidely. Indeed, the acquisition protocol of PPG-BP guarantees a rest period as well as a quiet and stable environement, which should provide more stable BP values and PPG signals.

The preprocessing screening criteria were devised to catch the most obvious signal issues, such as artefacts resulting from sudden movements or sensor disconnections, that could be seen in UCI. The exclusion thresholds were adjutsted incrementally to ensure that, through visual inspection of a sample of 100 UCI segments, only those with obvious issues were rejected. Thus, this step should not be seen as an optimized method of eliminating all possible segments with issues, but only those with the most flagrant signal quality issues. The aim was to remove those early in order to have less data to process and have a better baseline for statistical comparison for filtering the remaining, lesser issues, in later stages of processing. Segments are later excluded if fiducial points cannot all be extracted according to the constrains, or if the features produced are outliers.

The larger number of outliers in UCI compared to PPG-BP raises the question of whether those segments could be a result of extreme BP, and should not be rejected as outliers. It is however not the case. The DBP distribution of rejected segments is almost identical to that of retained segments. The SBP distribution is only slightly more skewed towards lower values for rejected segments with a mean and STD of 126.4 ± 21.8 mmHg compared to 131.8 ± 21.4 mmHg for retained segments. The large number of outliers can be explained by the lower signal quality of UCI, where noise and remaining artefacts can result in miss-detection of fiducial points or in abnormal pulse shapes, generating anomalous feature values.

Although not presented here, two pulse decomposition algorithms were evaluated to extract the *g* and *h* points: The recursive algorithm described by Kontaxis et al. (2020) and the gaussian fitting algorithm described by Couceiro et al. (2015). The first one gave very inconsistant results for pulses with different shapes, such as more pointed or wider top pulses and may not be appropriate to compare between subjects with such differences. R^2^ estimation results were also lower by as much as 0.18 with that method compared to the estimation method based on the second derivative described in Section 2.3. For the gaussian fitting method, R^2^ estimation results were similar while computation time for feature extraction was several times larger. The observation that some points in the second derivative were highly correlated with the position of the fitted gaussian components resulted in using those points directly, as described in Section 2.3.

To conclude, the various private datasets used in the indirect BP measurement literature make comparing the published algorithms difficult, and researchers have called for the creation of a standardised dataset suitable to compare and validate BP estimation algorithms (Solà and Delgado-Gonzalo, 2019). MIMIC, and by extension UCI, are publicly available and contain a large quantity of data, which may seem like a good basis for comparison. However, results presented in this paper reinforces suspicions of many researchers: that data sourced in intensive care units, under unknown conditions, may have a skewed response to BP and impair the generalisation of BP estimation algorithms. In fact, the issue of cross-dataset generalization is neither new nor limited to the field of BP estimation, but it is an issue often overlooked. It has been argued that cross-dataset validation of machine learning models developed for the medical field is essential to evaluate their performance (Thambawita et al., 2020). Yet, it is almost never done in the BP estimation literature. Cross-dataset generalisation can be challenging in itself, for example because of differences in equipment calibration, sampling, and recording conditions. Using intensive care data introduces an obvious sampling and recording condition bias. This is reflected in our presented results as significant differences in the relationship between BP and pulse features when comparing the UCI dataset to data obtained under more controlled conditions, which may make generalization more difficult to achieve. Besides using data that better represents the entire population, researchers could turn to data fusion and data augmentation to make their datasets more comprehensive. The latter has been used successfully in computer vision to improve cross-dataset performance, including in the field of imaging photoplethysmography (Nowara, 2021). In any case, we hope that the present paper raises awareness of this issue, replaces the vague suspicions surrounding intensive care data with quantified results that can be referred to, and stimulates better validations of models on different populations in future research.

## Data Availability

Publicly available datasets were analyzed in this study. This data can be found here: The UCI Cuffless Blood Pressure Estimation can be found here: https://archive.ics.uci.edu/ml/datasets/Cuff-Less+Blood+Pressure+Estimation. The PPG-BP dataset can be found here: https://figshare.com/articles/dataset/PPG-BP_Database_zip/5459299. The MIMIC 2 Waveform Database can be found here: https://archive.physionet.org/physiobank/database/mimic2wdb/.
